# Supplementary material for: Impact of aging and exercise on skeletal muscle mitochondrial capacity, energy metabolism, and physical function
Source: Nat Commun. 2021 Aug 6;12:4773. doi: 10.1038/s41467-021-24956-2 (PMC8346468; doi:10.1038/s41467-021-24956-2)
Supplement: Supplementary file 1 — Supplementary information [file 41467_2021_24956_MOESM1_ESM.pdf]

Supplementary notes

Comparable gait variability during unperturbed walking in older adults

Fourteen out of fifteen young participants completed all walking speed trials, with one person not able to walk at 1.8 m s^{-1} for two minutes. Twelve of seventeen old participants completed all speeds, with five unable to complete the 1.8 m s^{-1} speed. These differences between groups in the walking speeds generally align with the 6MWT results. When compared to young, older participants had a larger step length variability ($F_{(1, 30)} = 7.077$, $p = 0.012$; significant pairwise comparisons at 1.2, 1.4, and 1.6 m s^{-1}; Supplementary Figure 1), but no other significant differences were found.

Less gait stability but comparable adaptability during balance disturbances in older adults

All participants were able to recover from the gait perturbations without harness assistance. When considering group differences for the entire perturbation trial, the first perturbation ($Pert1_R$) had a significantly larger effect on the margin of stability (MoS) in the older adults compared to the young participants ($Pert1_R$: $F_{(1, 28)} = 7.7$, $p = 0.010$). For the second ($Pert2_L$) and ninth ($Pert9_L$) perturbations, no significant group differences were observed ($Pert2_L$: $F_{(1, 29)} = 0.6$, $p = 0.435$; $Pert9_L$: $F_{(1, 29)} = 0.001$, $p = 0.976$), indicating that older adults initially perform poorer than young adults, but the ability of older adults to adapt gait in response to repeated perturbations is intact. Furthermore, significant step by age group interaction effects on MoS were found for $Pert1_R$ and $Pert2_L$ but not $Pert9_L$ ($Pert1_R$: $F_{(9, 252)} = 6.3$, $p < 0.001$; $Pert2_L$: $F_{(9, 261)} = 3.539$, $p < 0.001$; $Pert9_L$: $F_{(9, 261)} = 1.413$, $p = 0.182$), indicative of different age-related recovery stepping behavior in the first two but not ninth perturbation. For all perturbations, the older adults' recovery steps, in comparison to the young adults, were more frequently different to Base and these differences also occurred in later recovery steps (significant results indicated by * in Supplementary Figure 2a).

When considering group differences at step level, the MoS in the first and second step after $Pert1_R$ and in the first step after $Pert2_L$ were observed to be significantly different between young and old (significant results indicated by # in Supplementary Figure 2a). These results indicate that the largest age group effect is seen in the first 1–2 recovery steps of the first two perturbations.

The young group required averages of 5.7 (1.8), 5.7 (1.6), and 3.6 (2.1) recovery steps and the older group required averages of 6.2 (2.4), 6.2 (2.1), and 4.2 (2.1) recovery steps for $Pert1_R$, $Pert2_L$, and $Pert9_L$, respectively (Supplementary Figure 2b). Both the young and older group required significantly fewer recovery steps during $Pert9_L$ than both $Pert1_R$ and $Pert2_L$ (Supplementary Figure 2b). No significant group differences were observed in the number of recovery steps for each perturbation.

Comparable gait variability during unperturbed walking in trained older adults

In O, twelve out of seventeen participants completed all speeds, with five participants unable to complete the 1.8 m s^{-1} speed. Of TO, all eighteen participants could complete all speeds. For IO, one participant completed all speeds, one completed all except 1.8 m s^{-1}, two reached 1.4 m s^{-1}, and one reached 1.2 m s^{-1}. These differences between groups in the walking speeds successfully achieved generally align with the 6MWT results. When comparing O and TO, no significant differences were observed for gait variability, including step time, step length, step width, and double support (Supplementary Figure 3).

A second set of similar analyses were conducted for the speeds 0.4 m s^{-1} up to 1.2 m s^{-1} including the O, TO, and IO groups. Here, a significant effect of group was found for double support time variability ($F_{(2, 37)} = 4.045$, $p = 0.0258$), with the IO group showing (non-significantly) more variability than the other two groups. No other group effects or pairwise comparisons were significant. Data shown in Supplementary Figure 4 also indicate non-significant increases in step width, step time variability, and (to a lesser extent) step length variability in IO. Together, the results indicate a less stable gait in IO, despite the insignificant findings due to the small sample size.

Comparable gait stability and adaptability in trained older adults

During the gait stability assessments, all measured participants were able to recover from the gait perturbations without harness assistance. When considering the entire perturbation trial (Supplementary Figure 5a), MoS was not significantly different between O and TO for $Pert1_R$, $Pert2_L$, and $Pert9_L$ ($Pert1_R$: $F_{(1, 32)} = 0,066$, $p = 0.8$; $Pert2_L$: $F_{(1, 32)} = 0.019$, $p = 0.892$; $Pert9_L$: $F_{(1, 33)} = 0.95$, $p = 0.338$). Furthermore, no significant step by group interaction effects on MoS were found for $Pert1_R$, $Pert2_L$, or $Pert9_L$ ($Pert1_R$: $F_{(9, 288)} = 0.62$, $p =0.784$; $Pert2_L$: $F_{(9, 288)} = 0.88$, $p = 0.544$; $Pert9_L$: $F_{(9, 297)} = 1.64$, $p = 0.102$). These results indicate no significant stability or stepping behavior difference between O and TO. No significant differences in MoS between the groups at step level were observed (Supplementary Figure 5a).

Following $Pert1_R$, $Pert2_L$, and $Pert9_L$, O required 6.2 (2.4), 6.2 (2.1), and 4.2 (2.1) recovery steps, respectively (Figure 13b). Following the same perturbations, TO required 6.1 (2.2), 5.2 (2.1), and 4.4 (2.5) recovery steps, respectively (Supplementary Figure 5b). O participants required significantly fewer recovery steps during $Pert9_L$ than both $Pert1_R$ and $Pert2_L$, while the TO adults required significantly fewer recovery steps during $Pert9_L$ than $Pert1_R$ (Supplementary Figure 5b). No significant group differences on number of recovery steps for each perturbation were revealed. Three participants of the IO group started and completed the perturbation trial, while three IO participants were unable to walk fast enough to maintain the stability-normalized walking speed. Therefore, they were not included in the previous

analyses. Following $Pert1_R$, $Pert2_L$, and $Pert9_L$, IO required 7.6, 6.0, and 4.6 recovery steps, respectively, with all three IO participants reducing their number of required recovery steps.

Supplementary Figures

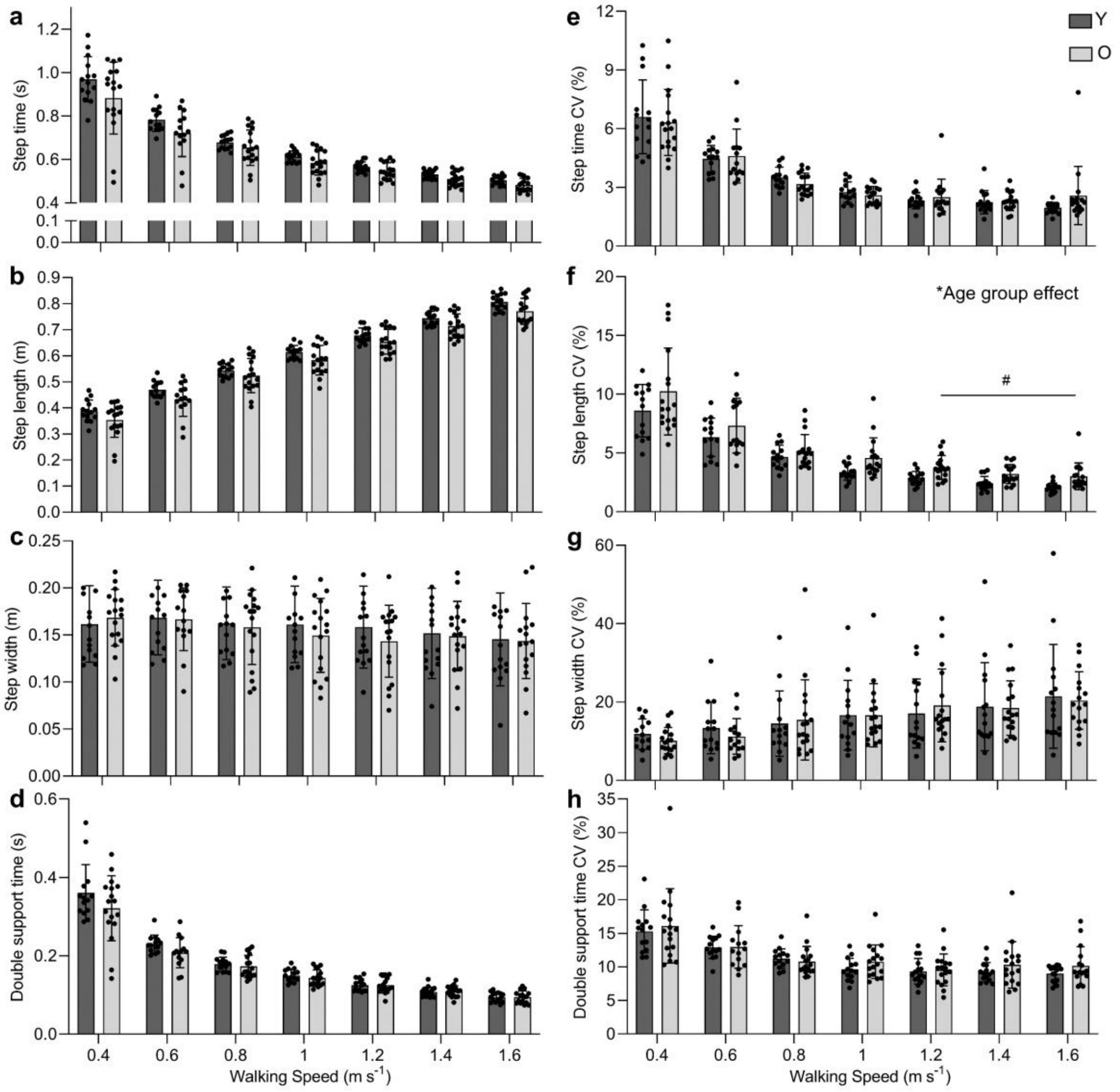

Supplementary Figure 1

Effect of aging on gait variability at different walking speeds (0.4–1.6 m s^{-1}). **a-d** Absolute values for step time, step length, step with, and double support time. **e-f** Coefficients of variation (CV) of step time, step length, step width, and double support time. Dark grey bars represent the young individuals (Y, n = 14); light grey bars represent older individuals (O, n = 17). One Y participant was excluded due to issues in data collection and two Y participants were not measured (one due to technical issues and one due to SARS-CoV-19 restrictions). Mixed-effects models using the restricted maximum likelihood method with age group and walking speed (repeated measure) as factors were conducted with pairwise Šídák's multiple comparisons test between groups at each speed. Values are presented as mean ± SD (with individual data points), * denotes age group effect denotes significant age group effect ($p < 0.05$) and # with line indicates pairwise differences between the two groups ($p < 0.05$).

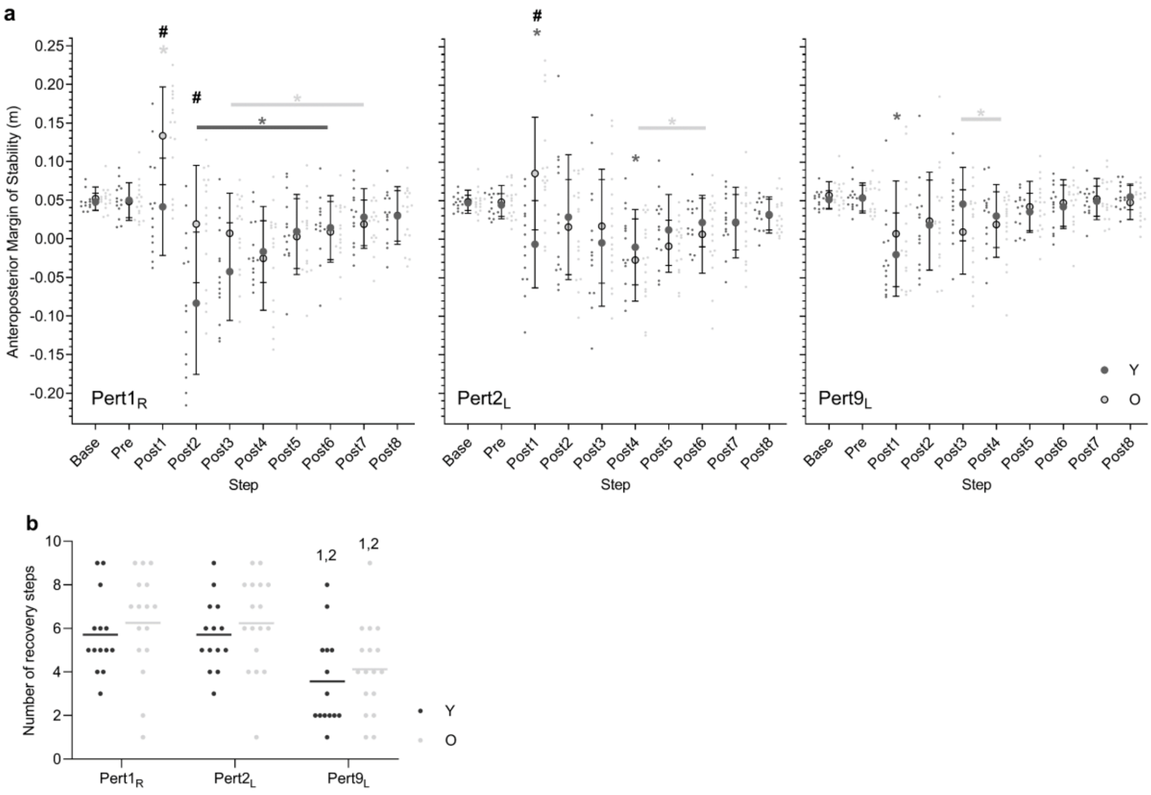

Supplementary Figure 2

Effect of aging on gait stability. **a** Mean and SD (with individual data points) of the anteroposterior margins of stability during the first, second, and ninth perturbations (Pert1$_R$, Pert2$_L$, and Pert9$_L$, respectively), including unperturbed walking prior to each perturbation (Base), the final step prior to each perturbation (Pre) and the first eight recovery steps following the perturbations (Post1 – 8) for young and older adults. Two-way repeated measures ANOVAs with group and step (repeated measures) as factors with within group pairwise comparisons to Base and between group pairwise comparisons were conducted. Dark grey* and light grey* indicate significant difference to Base for the young (Y, dark grey dots, n = 12) and older (O, light grey dots, n = 13) groups, respectively ($p < 0.05$, adjusted using Dunnett's multiple comparisons test). [#] indicates significant difference between the young and older groups ($p < 0.05$; adjusted using Šídák's multiple comparisons test). **b** The number of recovery steps (means and individual values) required by the young (dark grey dots, n = 12) and older adults (light grey dots, n = 13) for the first, second, and ninth perturbations (Pert1$_R$, Pert2$_L$, and Pert9$_L$, respectively). [1,2] indicate significant within-group difference to Pert1$_R$ and Pert2$_L$, respectively ($p < 0.05$, Wilcoxon matched-pairs signed rank tests). One Y participant was excluded due to an issue in data collection and two Y participants were not measured (one due to technical issues and one due to SARS-CoV-19 restrictions). Due to a technical issue with the treadmill, the data from the first perturbation of one of the O adults were excluded from the analysis.

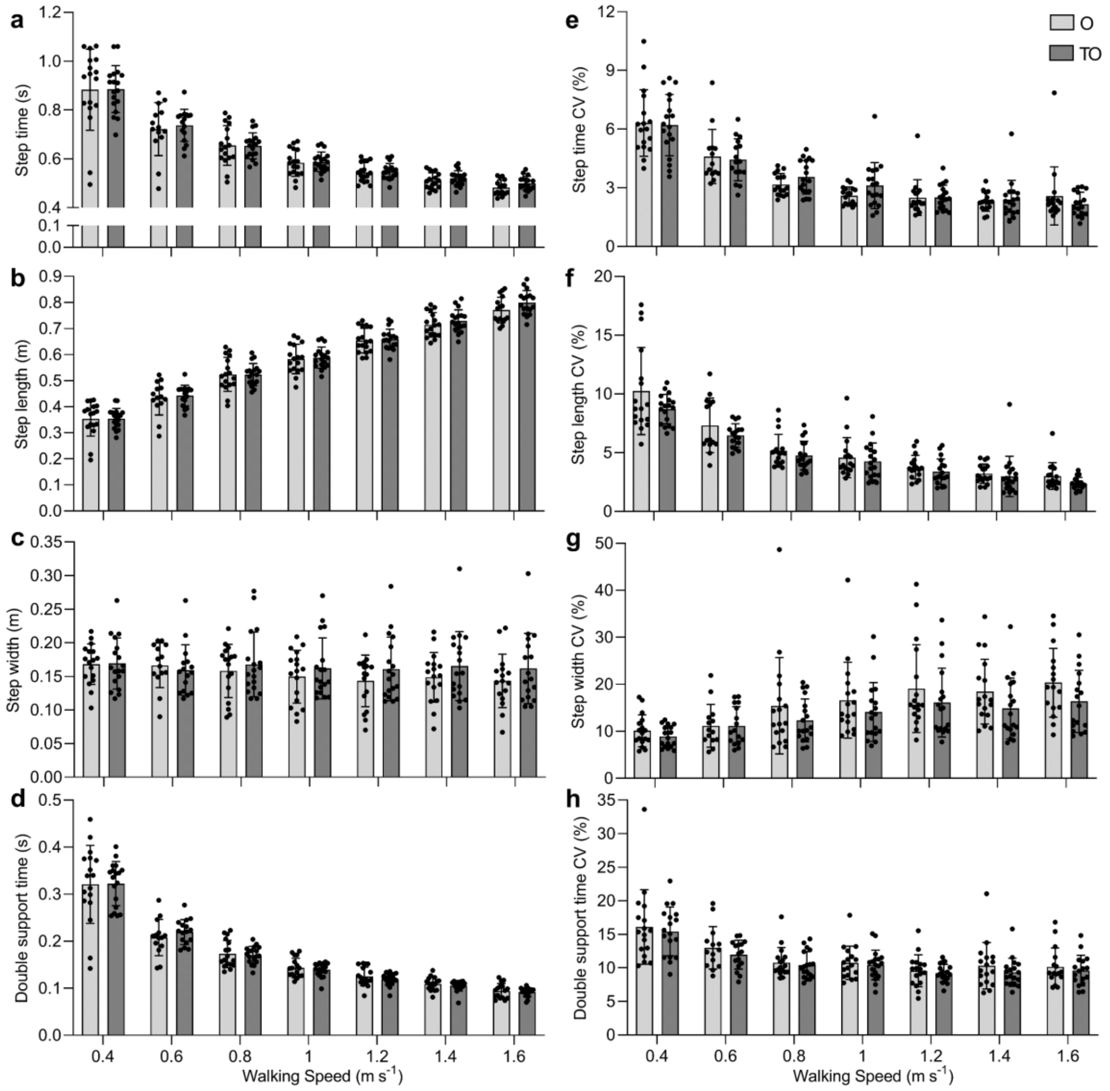

Supplementary Figure 3

Effect of exercise training on gait variability at different walking speeds (0.4–1.6 m s^{-1}). **a-d** Absolute values for step time, step length, step with and, double support time. **e-f** Coefficients of variation (CV) of step time, step length, step width, and double support time. Light grey bars represent normally active older adults (O, n = 17); dark grey bars represent trained older adults (TO, n = 18). One TO participant was not measured due to scheduling difficulties. Mixed-effects models using the restricted maximum likelihood method with group and walking speed (repeated measure) as factors were conducted with pairwise Šídák's multiple comparisons test between groups at each speed. Values are presented as mean ± SD (with individual data points).

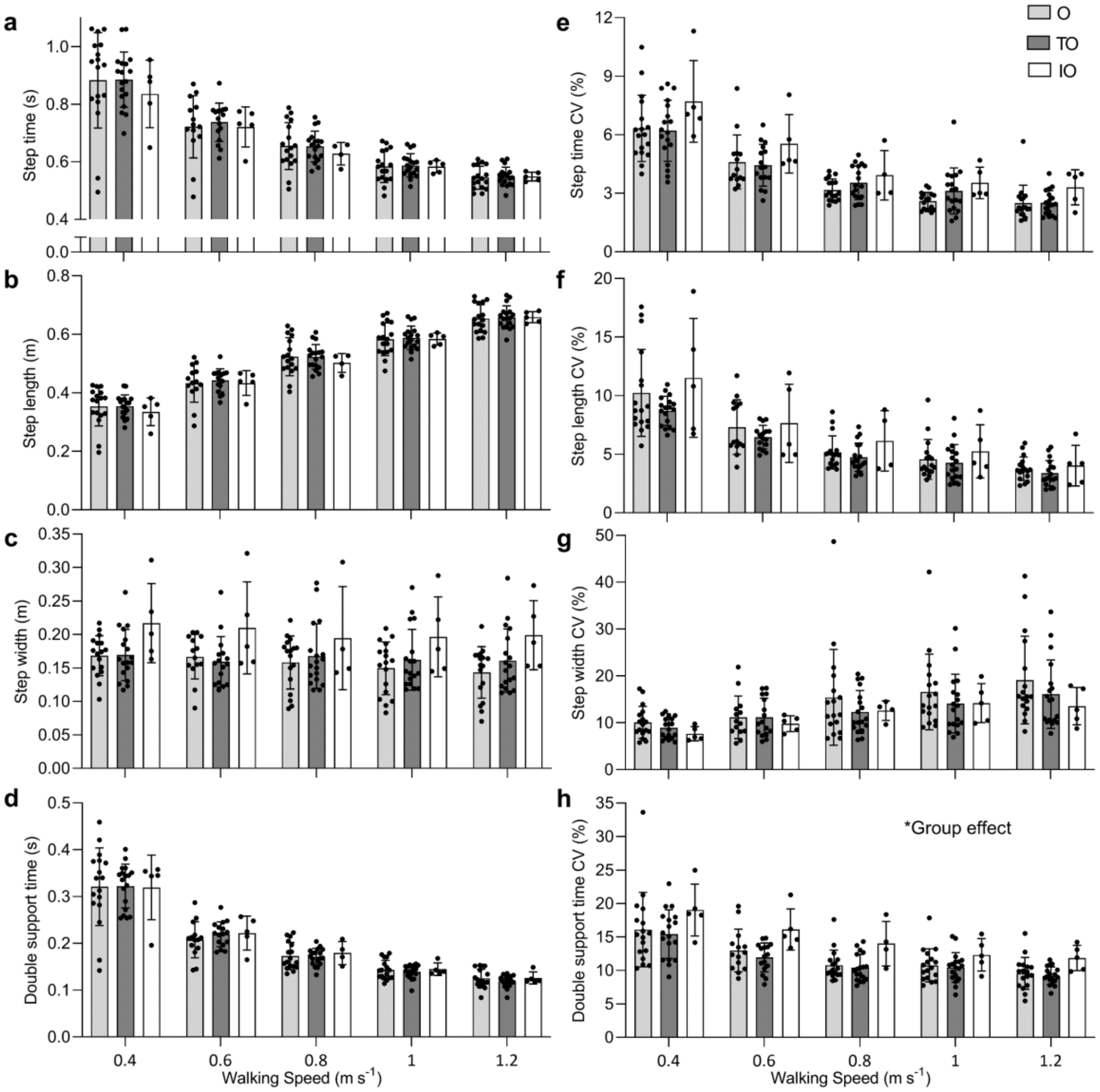

Supplementary Figure 4

Effect of exercise training and physical impairment on gait variability at different walking speeds (0.4–1.2 m s^{-1}). **a-d** Absolute values for step time, step length, step width, and double support time. **e-h** Coefficients of variation (CV) of step time, step length, step width, and double support time. Light grey bars represent normally active older adults (O, n = 17); dark grey bars represent trained older adults (TO, n = 18); white bars represent physically impaired older adults (IO, n = 5). One TO and one IO participant were not measured due to scheduling difficulties. Mixed-effects models using the restricted maximum likelihood method with group and walking speed (repeated measure) as factors were conducted with pairwise Šídák's multiple comparisons test between groups at each speed. Values are presented as mean ± SD (with individual data points), * denotes significant group effect ($p < 0.05$).

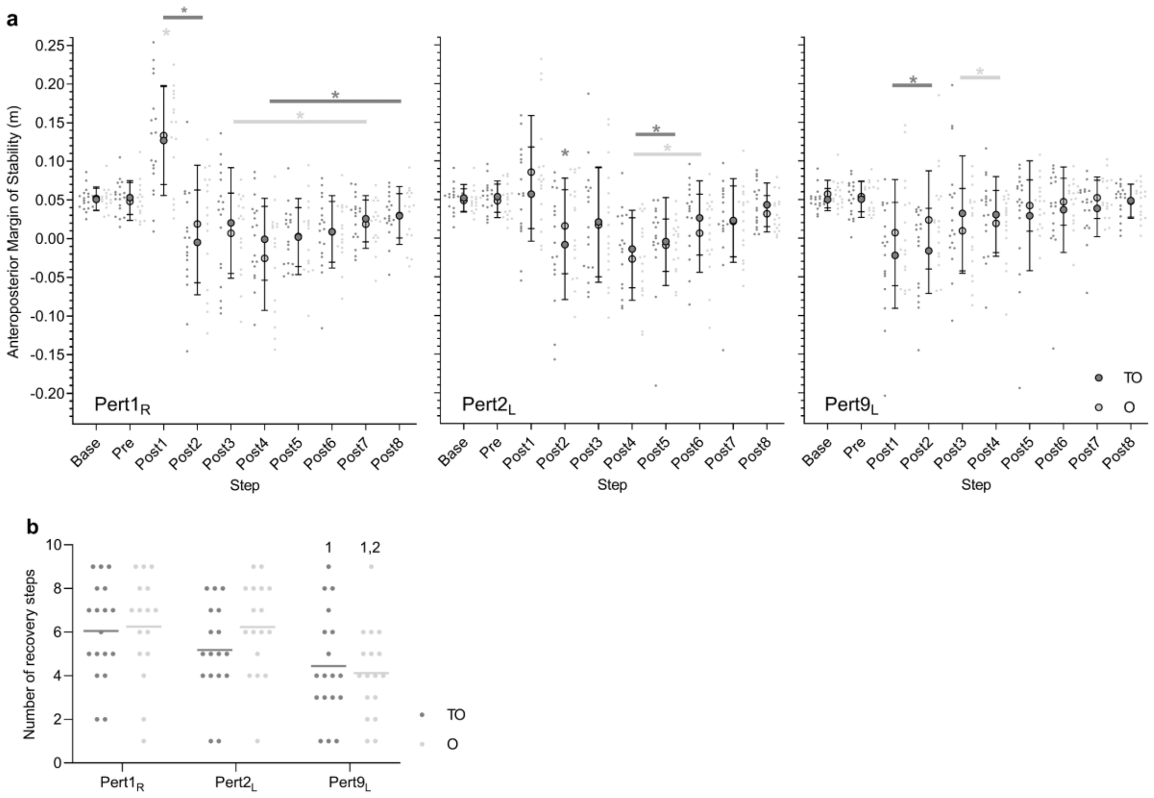

Supplementary Figure 5

Effect of exercise training on gait stability. **a** Means and ± SD (with individual data points) of the anteroposterior margins of stability during the first, second, and ninth perturbations ($Pert1_R$, $Pert2_L$, and $Pert9_L$, respectively), including unperturbed walking prior to each perturbation (Base), the final step prior to each perturbation (Pre) and the first eight recovery steps following the perturbations (Post1 – 8) for normally active older and trained older adults. Two-way repeated measures ANOVAs with group and step (repeated measures) as factors with within group pairwise comparisons to Base and between group pairwise comparisons were conducted. Dark grey * and light grey * indicate significant difference to Base for the O (light grey dots, n = 13) and TO (dark grey, dots n = 15) groups, respectively ($p < 0.05$, adjusted using Dunnett's multiple comparisons test). **b** The number of recovery steps (means and individual values) required by the normally active older (light grey dots, n = 13) and trained older adults (dark grey, dots n = 15) for the first, second, and ninth perturbations ($Pert1_R$, $Pert2_L$, and $Pert9_L$, respectively). [1,2] indicate significant within-group difference to $Pert1_R$ and $Pert2_L$, respectively ($p < 0.05$, Wilcoxon matched-pairs signed rank tests). One TO participant was not measured due to scheduling difficulties. Due to a technical issue with the treadmill, the data from the first perturbation of one of the O participants and from the second perturbation of one of the TO participants were excluded from the analyses. Only three individuals in the IO group were able to complete the perturbation trial, as such we compared only O versus TO.

Supplementary Tables

Supplementary Table 1 Study outline.

Screening	Visit 1<br>Week 1	Visit 2<br>Week 2	Visit 3<br>Week 3	Visit 4<br>Week 4	Visit 5<br>Week 5
Medical questionnaire	1-step hyperinsulinemic euglycemic	6MWT (Caren)	Body-composition (bodpod)	Resting metabolic rate	Muscle volume (MRI)
Physical examination		VO_2max cycle test	Gait variability and dynamic balance test (Caren)	Muscle biopsy	In vivo PCr recovery (^{31}P-MRS)
Physical function (SPPB)		^{31}P-MRS pre-test	Muscle strength (Biodex)	Ex vivo respirometry	
				Submaximal cycle test	

Visit #1 consisted of a 1-step hyperinsulinemic euglycemic clamp. On visit #2, participants completed a 6-minute walk test (6MWT) and a VO_2max measurement. Furthermore, an ActivPAL accelerometer was provided to determine free-living physical activity over a period of 5 days. On visit #3, participants completed a body composition measurement, a dynamic balance test, and quadriceps strength tests. On study visit #4, fasting blood samples were drawn, and resting energy expenditure was determined by indirect calorimetry followed by the muscle biopsy procedure. Participants then completed the submaximal exercise test on a cycle ergometer. On visit #5, participants completed the magnetic resonance imaging (MRI)/magnetic resonance spectroscopy (MRS)

Supplementary Table 2 Bivariate analysis to explore the relationships between energetics and physical muscle function.

Measurement	Variables		6MWT (m)	†Chair-stand test (s)	VO_2max (ml min^{-1} $kgFFM^{-1}$)	Isokinetic extension (Nm $kgFFM^{-1}$)	Isokinetic flexion (Nm kg FFM^{-1})	M-value (µmol kg^{-1} min^{-1})	S_i (µmol $kgFFM^{-1}$ min^{-1})	Daily steps (counts day^{-1})	#Recovery steps Pert1	Step length variability	Double support time step variability	Gross efficiency (%)
Mitochondrial function	Maximal coupled respiration MOGS3 (pmol s^{-1} mg^{-1})	r	0.335*	-0.111	0.436*	0.080	0.126	0.379*	0.174	0.326*	0.105	-0.177	-0.258	0.345*
		p	0.015	0.415	0.002	0.558	0.355	0.006	0.221	0.014	0.473	0.209	0.065	0.011
	Maximal uncoupled respiration (pmol s^{-1} mg^{-1})	r	0.310*	-0.146	0.512*	0.102	0.150	0.407*	0.232	0.372*	0.035	-0.228	-0.324*	0.402*
		p	0.025	0.282	<0.001	0.454	0.269	0.003	0.101	0.005	0.812	0.103	0.019	0.003
	PCr recovery rate constant k (s^{-1})	r	0.240	-0.075	0.482*	0.178	0.105	0.228	0.144	0.258	-0.124	-0.283*	-0.313*	0.315*
		p	0.090	0.587	0.001	0.193	0.444	0.108	0.318	0.057	0.402	0.044	0.025	0.021
Exercise efficiency	Gross efficiency (%)	r	0.679*	-0.485*	0.711*	0.349*	0.356*	0.495*	0.337*	0.268*	-0.368*	-0.277*	-0.193	
		p	<0.001	<0.001	<0.001	0.008	0.007	<0.001	0.015	0.046	0.008	0.044	0.166	
	Net efficiency (%)	r	0.596*	0.454*	0.623*	0.276*	0.261	0.480*	0.356*	0.207	-0.349*	-0.253	-0.249	
		p	<0.001	<0.001	<0.001	0.039	0.052	<0.001	0.009	0.127	0.013	0.068	0.072	

Data are from all groups (total n > 51). * indicates the correlation is significant at the 0.05 level (two-sided, $p < 0.05$); † indicates variables that were log transformed. MOGS3 = state 3 respiration upon malate + octanoyl-carnitine + glutamate + succinate; 6MWT = 6-minute walk test; VO_2max = maximal oxygen flow; FFM, = fat-free mass; Nm = newton meters; Pert1 = first perturbation during the balance challenge; M-value, = mean glucose infusion rate; S_i = insulin-stimulated glucose uptake; r = pearson or #spearman correlation coefficient as appropriate.
